# Dental Electricity

**Published:** 1901-07

**Authors:** 


### Bibliography.

DENTAL ELECTRICITY. By Levitt E. Custer, B.S., D.D.S., Lecturer upon Dental Electricity in Ohio College of Dental Surgery; Member of Röntgen Society of the United States, etc. Over two hundred illustrations. U. B. Publishing House, Dayton, Ohio, 1901.

This work on dental electricity does not seem appropriately named, as it gives the idea that the subject-matter of the book is confined to electrical apparatus necessary in dental operations, whereas it covers the most important facts in electrical science, and that in such a clear and comprehensive manner that it has impressed the reviewer as being one of the most satisfactory works for the dentist yet published.

The author says in his very brief preface, "Up to this time there is no single work to which the dentist might refer for information upon the electrical questions with which he has to deal without going over much that is foreign to the subject. . . . The author does not presume to present anything new upon the fundamental subjects, the nature of electricity, magnetism, etc., but rather in as concise a manner as possible to put them before the reader as a *résumé.* All technical terms are avoided save a few, without which even a cursory study of electricity would be wanting."

The latter fact furnishes the key that explains the value of the book for the unlearned in the science. It is, chapter by chapter, easily understood; indeed, becomes absorbingly interesting. While it is true that a third of the book is taken up with the elementary facts in electricity, yet these are treated in such a lucid manner, and so free from general technicalities, that the subject seems to assume a new and more vivid character. The author here shows the true teacher, the man of large experience in training others. The individual who writes without this almost invariably overloads his subject with technical terms, which not only obscure his meaning, but place an insurmountable barrier to the comprehension of the subject. Dr. Custer has happily avoided this, and hence his work is peculiarly well adapted to the minds it is supposed to educate.

The dentist of the present era has been thrust, as it were, into the midst of new conditions without the possibility of securing the necessary training to meet these properly. The introduction of electricity as a motive force has been so recent and so overwhelmingly rapid that the dental operator has been crushed by methods, terms, and appliances, with no possibility of securing a schooling that would fit him to understand the first, or skill, to the limit of safety, in the use of the latter.

The dental colleges of this country have, with very few exceptions, no regular study of this important science. The curriculum is now so overcrowded that teachers are unable to find room for anything further, and yet it is clearly evident that the management of the electric current must be understood if the dentistry of the future is to be properly regulated.

The older class of dental practitioners will be impressed, upon reading this book, with the wonderful progress made in the past few years in the invention of electrical dental appliances. Many of the methods of the past are now practically obsolete. The foot-power dental engine, the old-time furnace for porcelain, the annealing of gold, the drying of cavities, and a multitude of minor applications make the present period, as he reads, seem like the realization of something long hoped for but deemed impossible of attainment.

In view of the necessities of the dentist books have been prepared upon this science, but this one seems to meet more perfectly a general want, and, doubtless, will be used as a text-book in dental colleges and for constant reference by the general dental practitioner.

The book has been read, from cover to cover, with so much pleasure that it seems almost ungracious to allude to a small but positive defect, especially as the author has good ground for an expression of indignation. He states that his oven was patented to protect "the users . . . against rascally competition." Again, on the same subject, he speaks of one being "called to time." Further than this his personal allusions not only show temper, but will fail to strengthen the author's side of the argument, which, for those who know the facts, requires no doubtful support.

The dentist who follows the author carefully cannot fail to have made himself familiar with the foundation principles of electrical science, and no one not thus familiar has any warrant to make use of methods depending on the electric current, which when used understandingly is most efficient, but when used ignorantly may become dangerous.

The reviewer feels that the dental profession is under peculiar obligations to Dr. Custer, not only for his many inventions, but that he has given to dentistry his valuable experience in a permanent form. Those who may question the possibility of a dentist being an authority on this subject may be enlightened by knowing that the author has supplemented years of study by establishing an independent electrical plant to perfect his various appliances. He is recognized as an authority in electrical science, and his statements are to be accepted, therefore, with entire confidence.

A large sale is anticipated for this book, for the dentist of the future must use the electric current in some form. Every one, therefore, should be prepared to meet the changes that are already upon us, and which make a theoretical knowledge of electrical science an absolute necessity.

The general make-up of the book is exceptionally good, reflecting great credit on the U. B. Publishing House, Dayton, Ohio.

